# CO-19 PDB 2.0: A Comprehensive COVID-19 Database with Global Auto-Alerts, Statistical Analysis, and Cancer Correlations

**DOI:** 10.1093/database/baae072

**Published:** 2024-07-26

**Authors:** Shahid Ullah, Yingmei Li, Wajeeha Rahman, Farhan Ullah, Muhammad Ijaz, Anees Ullah, Gulzar Ahmad, Hameed Ullah, Tianshun Gao

**Affiliations:** S Khan Lab Mardan, Khyber Pakhtunkhwa, Pakistan; Department of Pharmacy, The Seventh Affiliated Hospital of Sun Yat-sen University, Shenzhen 518107, China; Big Data Center, The Seventh Affiliated Hospital of Sun Yat-sen University, Shenzhen 518107, China; S Khan Lab Mardan, Khyber Pakhtunkhwa, Pakistan; S Khan Lab Mardan, Khyber Pakhtunkhwa, Pakistan; S Khan Lab Mardan, Khyber Pakhtunkhwa, Pakistan; S Khan Lab Mardan, Khyber Pakhtunkhwa, Pakistan; S Khan Lab Mardan, Khyber Pakhtunkhwa, Pakistan; S Khan Lab Mardan, Khyber Pakhtunkhwa, Pakistan; Big Data Center, The Seventh Affiliated Hospital of Sun Yat-sen University, Shenzhen 518107, China

## Abstract

Biological databases serve as critical basics for modern research, and amid the dynamic landscape of biology, the COVID-19 database has emerged as an indispensable resource. The global outbreak of Covid-19, commencing in December 2019, necessitates comprehensive databases to unravel the intricate connections between this novel virus and cancer. Despite existing databases, a crucial need persists for a centralized and accessible method to acquire precise information within the research community. The main aim of the work is to develop a database which has all the COVID-19-related data available in just one click with auto global notifications. This gap is addressed by the meticulously designed COVID-19 Pandemic Database (CO-19 PDB 2.0), positioned as a comprehensive resource for researchers navigating the complexities of COVID-19 and cancer. Between December 2019 and June 2024, the CO-19 PDB 2.0 systematically collected and organized 120 datasets into six distinct categories, each catering to specific functionalities. These categories encompass a chemical structure database, a digital image database, a visualization tool database, a genomic database, a social science database, and a literature database. Functionalities range from image analysis and gene sequence information to data visualization and updates on environmental events. CO-19 PDB 2.0 has the option to choose either the search page for the database or the autonotification page, providing a seamless retrieval of information. The dedicated page introduces six predefined charts, providing insights into crucial criteria such as the number of cases and deaths’, country-wise distribution, ‘new cases and recovery’, and rates of death and recovery. The global impact of COVID-19 on cancer patients has led to extensive collaboration among research institutions, producing numerous articles and computational studies published in international journals. A key feature of this initiative is auto daily notifications for standardized information updates. Users can easily navigate based on different categories or use a direct search option. The study offers up-to-date COVID-19 datasets and global statistics on COVID-19 and cancer, highlighting the top 10 cancers diagnosed in the USA in 2022. Breast and prostate cancers are the most common, representing 30% and 26% of new cases, respectively. The initiative also ensures the removal or replacement of dead links, providing a valuable resource for researchers, healthcare professionals, and individuals. The database has been implemented in PHP, HTML, CSS and MySQL and is available freely at https://www.co-19pdb.habdsk.org/.

**Database URL**: https://www.co-19pdb.habdsk.org/

## Introduction

Biological databases serve as indispensable repositories that play a pivotal role in advancing scientific research and enhancing our understanding of complex biological systems [1–3]. These databases are critical for storing and organizing vast datasets, including DNA sequences, protein structures, and functional annotations [4]. They provide researchers with efficient access to biological information, enabling them to analyze and compare data effectively. Notable examples of such databases include GenBank [5], which specializes in genetic sequences, the Protein Data Bank (PDB) for protein structures [6], and UniProt [7], which offers comprehensive information on proteins. As our knowledge of biological processes continues to deepen, these databases evolve into dynamic tools that contribute significantly to genomics, proteomics, and bioinformatics.

Shifting our focus to the ongoing COVID-19 pandemic [8], the development of a Comprehensive COVID-19 Database represents a critical and timely initiative [9, 10]. This innovative platform integrates Global Auto-Alerts, Statistical Analysis, and an exploration of potential correlations with cancer. In the face of unprecedented challenges, this cutting-edge database stands as a beacon of hope, offering not just a centralized repository for COVID-19 data but also dynamic features for real-time tracking and insightful statistical analyses. As the world grapples with the multifaceted impacts of the pandemic, the need for a robust and integrated data management system becomes increasingly apparent [11]. At its core, the Comprehensive COVID-19 PDB Database serves as a meticulously designed centralized hub for collecting, managing, and disseminating critical information related to the pandemic [12]. The Global Auto-Alerts feature ensures that stakeholders, from healthcare professionals to policymakers, receive real-time updates on the latest developments. This proactive approach not only aids in the immediate response to emerging threats but also enhances the overall effectiveness of containment measures, thereby mitigating the impact of the virus on communities worldwide. One of the standout features of this database is its advanced Statistical Analysis capabilities. By harnessing the power of data analytics, the platform not only provides a comprehensive overview of the current state of the pandemic but also enables predictive modeling for future trends. This data-driven approach empowers researchers, epidemiologists, and public health officials to identify patterns, anticipate outbreaks, and strategically allocate resources. Moreover, the statistical insights generated by the platform contribute to a more nuanced understanding of the virus’s dynamics, fostering informed public discourse and guiding evidence-based policymaking. Beyond its immediate scope, the initiative takes a bold step toward unraveling potential correlations between COVID-19 and cancer. This unique exploration seeks to address the broader implications of the pandemic on global health. Given that both COVID-19 and cancer pose significant challenges to healthcare systems worldwide, understanding any potential interplay between the two becomes crucial. The platform’s ability to analyze and correlate data opens avenues for groundbreaking research, offering insights that could influence not only the ongoing pandemic response but also long-term strategies for tackling diverse health challenges. In practical terms, the Comprehensive COVID-19 Database’s Cancer Correlations feature delves into data points such as patient demographics, comorbidities, and treatment outcomes [13]. This nuanced approach to data analysis has the potential to revolutionize our comprehension of the virus, paving the way for targeted interventions, personalized healthcare strategies, and novel avenues for cancer research [13].

As the world grapples with the ongoing pandemic, the role of technology in healthcare takes the center stage [14]. The Comprehensive COVID-19 Database stands as a testament to the transformative power of data-driven solutions. Its holistic approach, integrating real-time alerts, statistical analysis, and a pioneering exploration into cancer correlations, positions it as a key player in the global effort to combat the pandemic and its far-reaching consequences. In a world where information is paramount, this initiative not only consolidates our understanding of the current crisis but also charts a course toward a future where data become an invaluable tool in safeguarding global health.

In conjunction with this groundbreaking initiative, the World Health Organization (WHO) plays a vital role in enhancing the quality of care for COVID-19 patients and survivors on a global scale [15, 16]. As new cases emerge worldwide, compiling data on recovery rates, new cases, deaths, and recovery rates becomes imperative [17, 18]. The distribution of cases by country reveals significant proportions, with the USA at 16.08%, India at 7.70%, Brazil at 5.86%, and France at 5.81%, among others. According to the WHO, the rates of all variant COVID-19 have decreased in almost all countries, underscoring the critical task of public health in 2022. Decreasing the COVID-19 rate necessitates a coordinated and comprehensive intervention across all facets of society, particularly the public sphere, civil society, and various professional sectors.

The correlation between cancer and COVID-19 is complex, reflecting both the susceptibility of cancer patients to severe outcomes from the virus and the impact of the pandemic on cancer care and outcomes. Studies have consistently shown that cancer patients are at higher risk of severe COVID-19 illness and mortality due to their weakened immune systems and underlying health conditions. According to research published in *The Lancet Oncology*, cancer patients infected with COVID-19 have a significantly higher mortality rate compared to the general population, with a reported case fatality rate ranging from 13% to 28%. Furthermore, disruptions in cancer screening, diagnosis, and treatment services during the pandemic have led to delays in care and adverse effects on cancer outcomes. A study by the American Cancer Society estimated that during the first 6 months of the pandemic, there was a 46% decline in cancer screenings compared to the previous year, potentially leading to delayed diagnoses and worsened prognoses for many patients. Thus, addressing the intersection of cancer and COVID-19 requires a multifaceted approach that prioritizes both the protection of cancer patients from the virus and the maintenance of essential cancer care services.

In summary, the Comprehensive COVID-19 Database, in collaboration with global efforts led by organizations such as the WHO, exemplifies the collaborative spirit required to combat the pandemic effectively [17, 18]. By leveraging technology, data analytics, and innovative approaches to understanding correlations, this initiative not only addresses the immediate challenges of the COVID-19 crisis but also lays the groundwork for a more resilient and proactive global health system in the face of future uncertainties. A total of 120 databases were identified, with meticulous attention given to removing any dead or broken links during the construction of the COVID-19 Pandemic Database (CO-19 PDB 2.0). The utilization of computer platforms such as PHP, HTML, CSS, and MySQL underscores the technical sophistication employed in this pioneering initiative. The comparison of CO-19 PDB 2.0 with other published works is shown in Table 1.

**Table 1.** Comparison of CO-19 PDB 2.0 with other published work

Authors	Year	Category	Format	DB. No.	Journal name	Ref.
CO-19 PDB 2.0	2024	COVID-19	DB + Table	120	*Database*	
Zou and Ma *et al*., 2015	2015	Human	Table	74	*Genomics, Proteomics Bioinf*.	[52]
Rigden and Fernández, 2021	2021	COVID + other	Table	89	*Nucleic Acids Res*.	[53]
Rigden and Fernández, 2020	2020	Nucleic acid	Table	70	*Nucleic Acids Res*.	[50]
Xu, 2012	2012	Protein	Table	121	*Curr. Protoc. Mol. Biol*.	[47]
Harper, 1994	1994	DNA + Protein	Table	50	*Curr. Opin. Biotechnol*.	[49]

## Materials and methods

### Database construction

**Figure 1.**
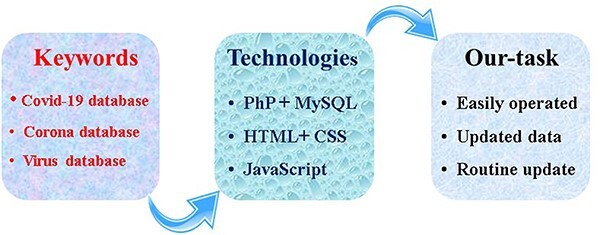
The data collection and construction procedure of CO-19 PDB Version 2.0 involved a meticulous and systematic process to ensure accuracy and reliability.

In crafting CO-19 PDB 2.0, the data collection and construction process prioritized user-friendliness and accessibility, as depicted in Fig. 1. To efficiently curate COVID-related data, a strategic approach involving various keywords like ‘databases of Covid-19,’ ‘COVID-19 pandemic database,’ ‘Coronavirus database,’ ‘Virus databases,’ and others was employed. This inclusive strategy aimed to cast a wide net for comprehensive data retrieval. Leading search engines such as Google Scholar, Google, and PubMed, along with reputable journals like *Nucleic Acids Research*, *Scientific Reports*, *Briefings in Bioinformatics*, and specialized database journals, were extensively utilized. A meticulous review identified 130 databases from existing sources, with particular attention given to eliminating any dead or broken links, ensuring the database’s reliability. The construction of CO-19 PDB 2.0 leveraged computer platforms including PHP, HTML, CSS, and MySQL, ensuring a robust and dynamic architecture for efficient data organization and presentation [12].

### Use of the CO-19 PDB 2.0

#### Searching the database

**Figure 2.**
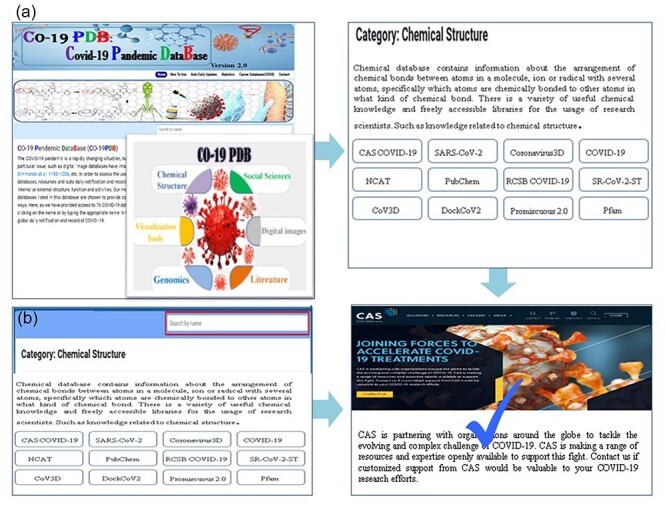
The utilization of CO-19 PDB is facilitated through a user-friendly interface, complemented by detailed stepwise images to enhance the user experience, (a) the search functionality, users can navigate by clicking on the category name, as illustrated in the provided insight; (b) users have the option to search for the required database by entering its name in the prominently highlighted search bar.

CO-19 PDB 2.0 offers users two convenient search options. First, users can explore the home page, selecting specific categories to access corresponding tables with concise descriptions (Fig. 2a). Further clicks direct users to the official link, accompanied by a brief query description. Upon clicking, a new window opens, presenting the required database, with a tick mark denoting its availability. Alternatively, users can use the search bar at the top of the main page (highlighted in Fig. 2b) to input the desired database name directly, facilitating efficient searching. The primary advantage of this database lies in its user-friendly design, allowing researchers to obtain their required data with a single click, eliminating the need for individual searches on platforms like Google. This not only saves researchers valuable time but also ensures that they access up-to-date information efficiently.

#### Daily and auto notification and record

**Figure 3.**
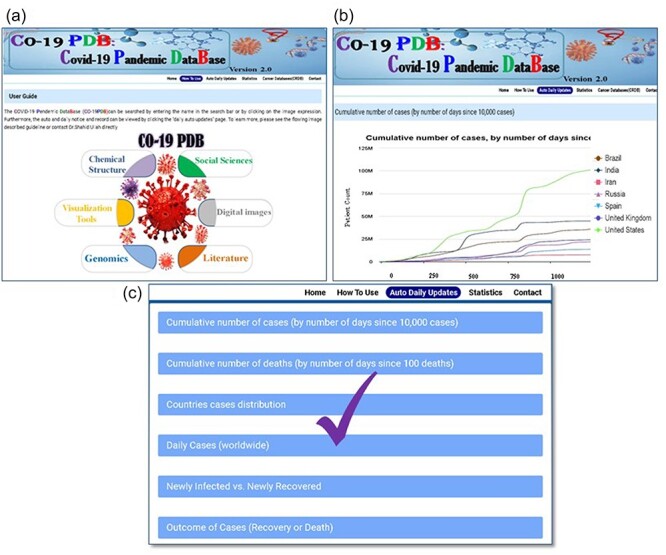
A thorough overview of the detailed methods for utilizing CO-19 PDB. (a) The user guidance page is a separate section that provides comprehensive details on using the database, offering insights into navigating the platform, and accessing information effectively. (b) This page provides comprehensive information on auto and daily updates. (c) The conclusive results and records of the gathered data and information.

CO-19 PDB 2.0 provides global auto and daily notifications and records for COVID-19. Users can access the user guide page for comprehensive information presented on the top bar of the database, as illustrated in Fig. 3a. Clicking on the ‘auto and daily updates’ page grants users access to all COVID-19 records and daily auto notifications, shown in (Fig. 3b). In Fig. 3c, we have highlighted six essential graphs among various others in the manuscript, ‘cumulate number of cases by number of day since 10000 cases’, ‘cumulate number of deaths by number of days since 100 deaths’, ‘countries cases distribution’, ‘daily cases worldwide’, ‘Newly Infected vs. Newly Recovered’, and ‘Outcome of Cases (Recovery or Death)’.

## Results

### Database statistics

**Figure 4.**
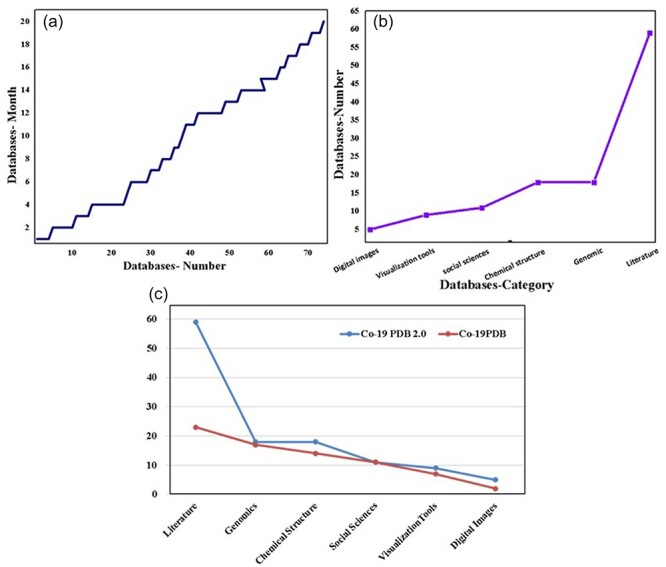
The complete statistics of CO-19 PDB 2.0, including (a) month-wise growth of the COVID databases, (b) category-wise growth, and (c) the number and comparison of entries between the previous database and the updated version (CO-19 PDB 2.0).

In this study, our primary focus is on COVID-19 databases, encompassing data gathered from December 2019 to April 2023. The graphical representation in Fig. 4a vividly displays the monthly surge in databases, showcasing a notable increase over the specified period. Figure 4b further delineates this growth, categorizing databases by their types. Notably, the literature category stands out with the highest value, reflecting the substantial volume of globally published COVID-19 literature. Figure 4c provides a comprehensive analysis of the data, including a detailed breakdown and comparison with previously published data, which is essential for future studies. In the figure, the red bars denote the previous data, while the blue bars represent the updated information from CO-19 PDB 2.0. The literature category takes the lead with 59 entries, followed by the genome and chemical structure categories, each with 18 entries. To ensure the ongoing relevance of our findings, a dedicated portal for the most recent information and research has been established. Importantly, we have meticulously updated or removed redundant, disabled, and inaccessible database links. Additionally, new and updated Covid-19 databases have been incorporated, presented in both tabular and database format (Supplementary Table S1). This approach ensures the accessibility and accuracy of the compiled information for researchers and practitioners alike.

### Global Covid patients and death

**Figure 5.**
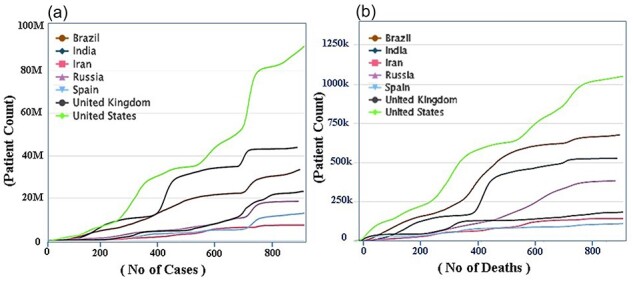
A detailed overview of the country-wise statistics of COVID-19. (a) The statistics on the number of patients and new cases during the COVID-19 period and (b) The data on the number of deaths and the recovery rate.

According to WHO for COVID research [19], the rates of all COVID variants have decreased in almost all countries, the privation of which is the major task for public health in 2022 [20, 21]. Decreasing the COVID rate involves coordinated and comprehensive intervention from all facets of society, particularly the public sphere, civil society, and health and other occupations. Figure 5 shows the number of cases number of death and total patent countries wisely, Fig. 5a shows the number of patients and new cases, in which USA is on the top (92 Million) and India is on the second (40 Million), Brazil, UK, Iran, and so on respectively, while the updated data is available on CO-19 PDB 2.0 site, the stander is kept ‘Cumulative number of cases (by number of days since 10 000 cases’. Although the mortality estimates and patients compression are shown in Fig. 5b, which is the cumulative number of deaths (by number of days since 100 deaths), the USA is on the top (1 049 274) Brazil is on the 2nd (675 353), India is on 3rd position (525 474) and so on the new updated data can be views on https://www.co-19pdb.habdsk.org/daily-updates links.

### New cases and deaths

**Figure 6.**
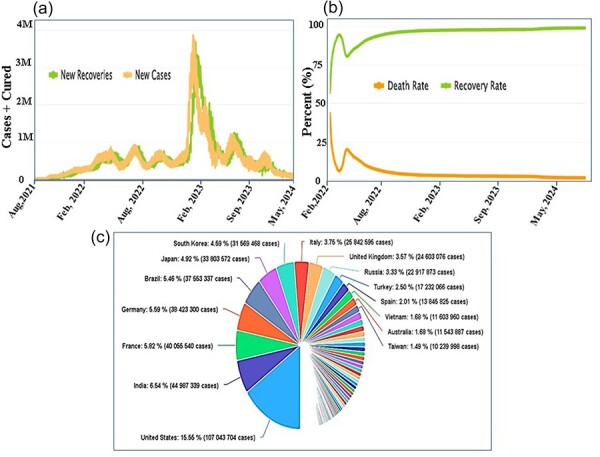
Detailed daily and auto statistic information on COVID. (a) Daily updates on the rates of new recoveries and new cases among COVID-19 patients, (b) a comprehensive overview of the details regarding the death and recovery rates, and (c) a detailed breakdown of the country-wise distribution.

The WHO operates globally to enhance the quality of care for COVID patients and survivors. Over time, amid the evolving global scenario, we have compiled comprehensive data on new recoveries, new cases, death rates, and recovery rates, allowing for a detailed comparison. In Fig. 6a, the daily dynamics of newly infected individuals versus recoveries are illustrated. The brown curve highlights the surge in new cases, peaking at 4 million in January 2022 to February 2020, while the green curve showcases a rapid recovery trend, particularly noticeable from April onward. Figure 6b presents the cumulative total deaths versus recoveries, indicating a declining death rate (brown) since February 2022 and a significant recovery rate (green). The country-wise distribution in Fig. 6c reveals percentages, with the USA at 16.08%, India at 7.70%, Brazil at 5.86%, France at 5.81%, and others as detailed in the figure. For real-time updates, the data can be accessed by clicking the following link: https://www.co-19pdb.habdsk.org/daily-updates. This comprehensive analysis offers valuable insights into the global Covid scenario, aiding in informed decision-making and public health strategies.

### New cancer cases and deaths

**Figure 7.**
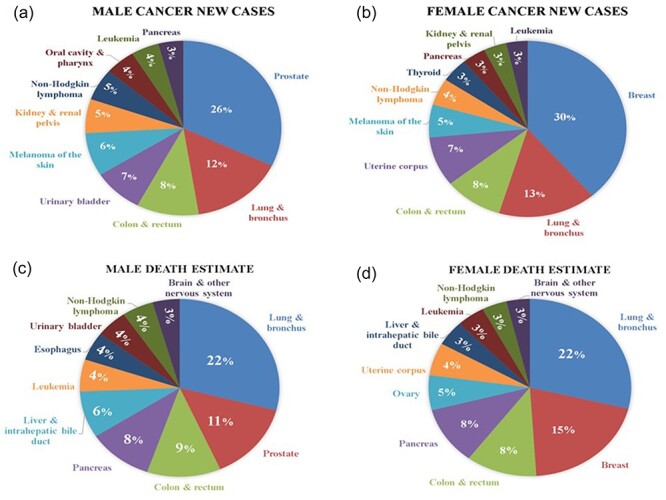
The number of new cancer cases and mortality rates during the COVID-19 period. (a and b) The number of new cancer cases among both males and females during the COVID period and (c and d) detailed insights into the mortality estimates among both males and females during the COVID-19 period.

According to Cancer Figure and Facts 2024 [22], The American Cancer Society Cancer Action Network is dedicated to improving the quality of care for cancer victims and survivors on a global scale [13]. In the context of the ongoing COVID-19 pandemic, we have examined and compiled data on the top 10 cancers diagnosed in the USA in 2024. According to the findings presented in Fig. 7a and b), breast cancer in women and prostate cancer in men are the most prevalent, accounting for 30% and 26% of new cancer cases, respectively. Furthermore, mortality estimates during the COVID-19 period are depicted in Fig. 7c and d. Interestingly, lung and bronchus cancer emerge as the highest cause of mortality in both male and female patients, followed by breast and prostate cancers in the second position. These detailed insights contribute to a better understanding of the impact of the COVID-19 era on cancer incidence and mortality rates, providing valuable information for healthcare professionals and policymakers alike.

## Discussion

### CO-19 PDB classification

The categorization of diverse viruses relies on discerning features, enabling their differentiation [23].This differentiation is accomplished through the assessment of sequence similarity [24], examination of the molecular structure of the genome [25], consideration of pathogenicity [26], analysis of structural similarities [27, 28], usage of phytochemicals [29], and evaluation of the host range [30]. Extensive research on various viruses has resulted in a wealth of information stored in the published literature and databases for convenient access. Notable databases like National Institute of Health, the COVID-19 Data Portal, and Emergency DataBase of COVID-19 have been established, utilizing these vast data. Amid the ongoing COVID-19 pandemic, numerous databases have surfaced. However, there remains a necessity for a user-friendly and simplified research platform catering to the scientific community’s needs. To address this, we have curated and organized revised datasets into distinct categories based on their external and internal structures and functions. These resources are accessible through the following link: https://www.co-19pdb.habdsk.org/, providing a streamlined avenue for the scientific community to access relevant and comprehensible COVID-19 research.

### Chemical structure database

The COVID Chemical Structure Database is a vital resource that catalogs molecular structures crucial for virus research [31]. This repository compiles detailed information on chemical components, aiding research into therapeutic interventions, drug development, and a deeper understanding of the virus at the molecular level [32]. Offering a comprehensive collection of chemical structures associated with COVID-19, the database becomes an invaluable tool for scientists exploring novel compounds with antiviral properties. Access to this repository amplifies the investigation of essential chemical interactions, crucial for advancing treatments and mitigating the impact of the pandemic.

### Visualization tool database

Visualization tools are crucial for translating intricate datasets into understandable insights within databases. Platforms such as Tableau [33], Power BI [34], and Google Data Studio [35] exemplify these tools, empowering users to convert raw data into visually compelling charts, graphs, and dashboards. The graphical representation not only simplifies the comprehension of complex patterns but also enriches the interpretability of data trends. With interactive features and dynamic interfaces, these tools facilitate a more immersive and intuitive exploration of information. In the current era of data-driven decision-making, visualization tools act as a vital link between raw data and actionable insights, enabling businesses and individuals to make well-informed choices based on a comprehensive understanding of their data landscape. As organizations increasingly prioritize visual communication of data, these tools become indispensable assets, driving effective analysis, communication, and decision-making processes [36]. ‘CDC’ represents the newly introduced COVID-19 Community Levels tool. This tool serves as a valuable resource, aiding individuals and communities in determining appropriate prevention measures based on the prevailing rates of hospitalizations and cases [37].

### Genomic database

The COVID Genomic Database is a pivotal tool for unraveling the genetic intricacies of the SARS-CoV-2 virus. This database systematically compiles and analyzes genomic sequences, fostering global research on the virus’s evolution, variants, and potential implications for public health. By documenting and comparing genetic data, scientists can monitor mutations, track the emergence of new variants, and inform strategies for diagnostics, treatments, and vaccine development. Positioned at the forefront of molecular research, the COVID Genomic Database significantly contributes to our understanding of the virus, enabling a more nuanced and adaptive response to the ever-evolving landscape of the COVID-19 pandemic [38]. “Pager–COV” is a database presenting pathways and gene lists linked to COVID-19. This resource offers vital insights for researchers and clinicians studying the virus’s intricacies, providing comprehensive information on molecular pathways and relevant genes, facilitating a deeper understanding of COVID-19’s impact on cellular processes.

### Social science database

The COVID Social Science Database stands as a crucial repository amid the ongoing global pandemic, consolidating a diverse array of research spanning sociology, psychology, economics, and public health [39, 40]. This comprehensive resource facilitates a nuanced understanding of COVID-19’s profound social impact, catering to the needs of researchers, policymakers, and the general public [41]. Delving into changes in social structures, mental health implications, economic trends, and regional variations, the database offers valuable insights into societal responses. Beyond its role as a static collection, it actively shapes future research by identifying gaps and evolving in tandem with the pandemic’s developments. In a time where the social dimensions of the crisis are as vital as medical considerations, this database becomes an indispensable tool, promoting interdisciplinary collaboration and contributing to informed decision-making for the establishment of resilient and adaptive societies. The “EU Portal” is a web dataset containing the latest public data on COVID-19. It includes a daily situation update, the epidemiological curve, and global geographical distribution for the European Union/European Economic Area, the UK, and worldwide. This platform offers concise and current information on the pandemic’s status, providing insights into daily updates, epidemiological trends, and the global spread of the virus.

### Digital image database

The COVID Digital Image Database stands as a vital asset in the global fight against the pandemic, functioning as an extensive archive of medical images associated with COVID-19 [42]. This digital repository assumes a pivotal role in propelling research, refining diagnostic methodologies, and optimizing treatment strategies by offering a varied assortment of radiological images, encompassing X-rays and CT scans [43, 44]. These images vividly illustrate the virus’s manifestations in afflicted individuals. This resource empowers researchers and healthcare professionals to discern patterns, scrutinize disease progression, and devise more efficient diagnostic instruments. Beyond fostering collaboration among medical experts, the COVID Digital Image Database expedites innovation in the relentless battle against the pandemic, enhancing our comprehension of COVID-19 and ultimately elevating patient outcomes. Among all “COVID Digital Pathology Repository,” or the COVID Digital Pathology Repository, emerges as a vital resource bolstering the biomedical community in our collective response to the COVID-19 virus pandemic.

### Literature database

The COVID Literature Database serves as a pivotal nexus for consolidating and disseminating a diverse array of research, studies, and scholarly articles pertaining to the COVID-19 pandemic [45]. This extensive repository is instrumental in advancing scientific knowledge, providing a centralized platform for researchers, healthcare professionals, and policymakers to access the latest insights. Spanning various disciplines, from medicine and epidemiology to the social sciences, the database enables a nuanced understanding of the virus’s multifaceted impacts. Researchers utilize this resource to stay current with evolving trends, shape evidence-based decision-making, and foster collaborative endeavors in the global battle against COVID-19. As a crucial instrument for navigating the complex landscape of pandemic-related information, the Covid Literature Database contributes significantly to cultivating informed responses and implementing effective public health strategies. The “COVID-19 GPH” initiative is a case in point; the pandemic has expedited the integration of digital health in India and other regions [46]. For instance, in New Delhi, a telemedicine hub has emerged, symbolizing the broader trend.

### Advantage of this work

The multifaceted advantages of the CO-19 PDB 2.0 initiative are profound and transformative for the global research community. Historically, numerous articles and biological databases have been compiled, recognizing the critical role of databases in modern biology [47–50]. With the emergence of the COVID-19 pandemic, the CO-19 PDB 2.0 addresses a crucial need for a centralized, accessible, and user-friendly resource to unravel the intricate connections between the novel virus and cancer [51]. Its comprehensive nature is underscored by meticulous categorization into six distinct functionalities, offering datasets that range from chemical structures and digital images to genomics and social sciences. The initiative not only introduces auto daily notifications but also presents an intuitive navigation system, predefined charts, and a direct search option, ensuring seamless information retrieval. The collaborative effort with 90 recognized research institutions enhances its global impact, generating a wealth of research articles and computational work documented in reputable international journals. By emphasizing the correlation between COVID-19 and cancer, including the top 10 cancers diagnosed in the USA in 2022, the initiative provides invaluable insights. The removal of dead links and the use of a robust technology stack contribute to the initiative’s reliability and efficiency. Beyond compiling up-to-date datasets, this comprehensive endeavor serves as a crucial resource for researchers, healthcare professionals, and individuals navigating the intricate landscape of COVID-19 data within the context of cancer, thereby fostering extensive sharing of open data and benefiting global research communities.

### Limitation of the work

We have developed a well-organized COVID-19 database and resources to provide information more efficiently. Our work is freely available for all academic research under the journal’s rules and limitations.

## Conclusion

The worldwide ripple effect of COVID-19 has disproportionately impacted individuals contending with cancer, intensifying their vulnerability. Presently, 90 globally recognized research institutions actively engage with the multifaceted challenges of COVID-19, with some specifically scrutinizing its correlation with cancer. This collaborative effort has yielded a wealth of documented research articles and computational work, published in reputable international journals. This platform introduces a groundbreaking feature—auto daily notifications—presenting information in a standardized format. Key metrics, including “number of cases and deaths,” country-wise distribution, “new cases and recovery,” and daily updates on death and recovery rates, are provided in a unique and accessible manner. Navigation is designed to be intuitive, offering users the option to explore categories based on physical and chemical features, enhancing overall user experience. A search option streamlines information retrieval, allowing users to enter the name of the required database directly. Our study not only compiles up-to-date COVID-19 datasets but also presents global statistics on the correlation between COVID-19 and cancer. Emphasis is placed on the top 10 cancers diagnosed in the USA in 2022, revealing breast cancer in women and prostate cancer in men as the most prevalent, constituting 30% and 26% of new cancer cases, respectively. Mortality estimates during COVID underscore the prominence of lung and bronchus cancers, affecting both genders equally, with breast and prostate cancers following closely. This comprehensive initiative prioritizes updated and reliable information by removing or replacing all dead and broken links. By providing a holistic view of the COVID and cancer correlation, our endeavor serves as an invaluable resource for researchers, healthcare professionals, and individuals navigating the intricate landscape of COVID-19 data within the context of cancer.

## Supplementary Material

baae072_Supp

## Data Availability

All relevant data are included in the paper and can be downloaded directly from the database using the provided link. Additionally, the data will be available according to the journal’s rules and regulations.
